# Case Series of Multisystem Inflammatory Syndrome in Adults Associated with SARS-CoV-2 Infection — United Kingdom and United States, March–August 2020

**DOI:** 10.15585/mmwr.mm6940e1

**Published:** 2020-10-09

**Authors:** Sapna Bamrah Morris, Noah G. Schwartz, Pragna Patel, Lilian Abbo, Laura Beauchamps, Shuba Balan, Ellen H. Lee, Rachel Paneth-Pollak, Anita Geevarughese, Maura K. Lash, Marie S. Dorsinville, Vennus Ballen, Daniel P. Eiras, Christopher Newton-Cheh, Emer Smith, Sara Robinson, Patricia Stogsdill, Sarah Lim, Sharon E. Fox, Gillian Richardson, Julie Hand, Nora T. Oliver, Aaron Kofman, Bobbi Bryant, Zachary Ende, Deblina Datta, Ermias Belay, Shana Godfred-Cato

**Affiliations:** ^1^CDC COVID-19 Response Team; ^2^Epidemic Intelligence Service, CDC; ^3^University of Miami Miller School of Medicine and Jackson Health System, Florida; ^4^New York City Department of Health and Mental Hygiene; ^5^Massachusetts General Hospital, Boston, Massachusetts; ^6^Broad Institute of MIT and Harvard, Cambridge, Massachusetts; ^7^Maine Center for Disease Control and Prevention; ^8^University of Southern Maine, Portland, Maine; ^9^Maine Medical Center/Maine Medical Partners, Portland, Maine; ^10^Minnesota Department of Health; ^11^Lousiana State University Health Sciences Center, New Orleans, Louisiana; ^12^Southeast Louisiana Veterans Healthcare System, New Orleans, Louisiana; ^13^Louisiana Department of Health; ^14^Section of Infectious Diseases, Atlanta VA Medical Center, Decatur, Georgia; ^15^Division of Infectious Diseases, Emory University School of Medicine, Atlanta, Georgia; ^16^Oak Ridge Institute for Science and Education, Oak Ridge, Tennessee.

During the course of the coronavirus disease 2019 (COVID-19) pandemic, reports of a new multisystem inflammatory syndrome in children (MIS-C) have been increasing in Europe and the United States (*1*–*3*). Clinical features in children have varied but predominantly include shock, cardiac dysfunction, abdominal pain, and elevated inflammatory markers, including C-reactive protein (CRP), ferritin, D-dimer, and interleukin-6 (*1*). Since June 2020, several case reports have described a similar syndrome in adults; this review describes in detail nine patients reported to CDC, seven from published case reports, and summarizes the findings in 11 patients described in three case series in peer-reviewed journals (*4**–**6*). These 27 patients had cardiovascular, gastrointestinal, dermatologic, and neurologic symptoms without severe respiratory illness and concurrently received positive test results for SARS-CoV-2, the virus that causes COVID-19, by polymerase chain reaction (PCR) or antibody assays indicating recent infection. Reports of these patients highlight the recognition of an illness referred to here as multisystem inflammatory syndrome in adults (MIS-A), the heterogeneity of clinical signs and symptoms, and the role for antibody testing in identifying similar cases among adults. Clinicians and health departments should consider MIS-A in adults with compatible signs and symptoms. These patients might not have positive SARS-CoV-2 PCR or antigen test results, and antibody testing might be needed to confirm previous SARS-CoV-2 infection. Because of the temporal association between MIS-A and SARS-CoV-2 infections, interventions that prevent COVID-19 might prevent MIS-A. Further research is needed to understand the pathogenesis and long-term effects of this newly described condition. 

Potential MIS-A patients were identified from several sources: reports from clinicians and health departments, published case reports, and published case series. Clinicians and health departments in the United States voluntarily reported adult patients with suspected MIS-A to CDC using the case report form* developed for MIS-C after a Health Advisory was published on May 14, 2020, calling for reporting of MIS-C cases. The case report form included information on patient demographics, underlying medical conditions, clinical findings, complications, laboratory test results including those from SARS-CoV-2 testing, imaging findings, treatments, and outcomes. Two clinician reviewers selected patients who fulfilled the working MIS-A case definition used in this report, which included the following five criteria: 1) a severe illness requiring hospitalization in a person aged ≥21 years; 2) a positive test result for current or previous SARS-CoV-2 infection (nucleic acid, antigen, or antibody) during admission or in the previous 12 weeks; 3) severe dysfunction of one or more extrapulmonary organ systems (e.g., hypotension or shock, cardiac dysfunction, arterial or venous thrombosis or thromboembolism, or acute liver injury); 4) laboratory evidence of severe inflammation (e.g., elevated CRP, ferritin, D-dimer, or interleukin-6); and 5) absence of severe respiratory illness (to exclude patients in which inflammation and organ dysfunction might be attributable simply to tissue hypoxia). Patients with mild respiratory symptoms who met these criteria were included. Patients were excluded if alternative diagnoses such as bacterial sepsis were identified.

To identify potential published cases, a literature search was performed on August 20, 2020, and 355 publications were identified.^†^ Abstracts were screened by one reviewer to determine whether cases met the working MIS-A case definition; when no abstract was available, the full paper was examined. The references were reviewed to identify additional relevant articles. Data were obtained from published reports; authors were contacted to confirm published data and, when necessary, to provide data not included in the original articles.

## Case Reports

**Demographic characteristics and underlying conditions**. Cases in nine patients reported to CDC (Table 1) and seven published case reports (Table 2), originating from seven U.S. jurisdictions and the United Kingdom, met the working case definition. The 16 patients ranged in age from 21 to 50 years and included seven men and nine women. Five were reported as Hispanic, nine as African American, one as Asian, and one as a United Kingdom–born man of African ethnicity. Nine patients had no reported underlying medical conditions; six were obese, one had poorly controlled diabetes mellitus type 2 (hemoglobin A1C >9.0%), two had hypertension, and one had obstructive sleep apnea. Eight patients had documented respiratory illness before developing symptoms of MIS-A, and eight did not.

**TABLE 1:** Demographics, clinical features, treatments, and outcomes of nine adults reported to CDC with multisystem inflammatory syndrome (MIS) associated with SARS-CoV-2 infection — United States, March–August 2020

Age (yrs), sex, race/ethnicity, location	Underlying medical conditions	Clinical signs and symptoms	Previous respiratory illness/SARS-CoV-2 testing	SARS-CoV-2 testing at time of MIS-A admission	Laboratory studies (peak)*	Imaging/Other diagnostic studies	Treatments	Outcome and length of stay
Patient 1: 27, female, African American, Maine	None	Rigors, profuse diarrhea, diffuse rash x 5 days. Admitted with mixed shock (hypovolemic, vasoplegic, cardiogenic) and acute renal failure.	No/Testing unknown	PCR (-), Ab (+)	CRP 344 mg/L; D-dimer 2818 ng/mL; ferritin 1082 ng/mL; troponin I 0.43 ng/mL; ALT 37 IU/L; ALC nadir 420 cells/μL	TTE: mild to moderate global hypokinesis, left ventricular ejection fraction 45%, mildly dilated right ventricle, mild tricuspid regurgitation, pericardial effusion. CT chest: bilateral patchy ground-glass opacities, pleural effusion. CT abdomen/pelvis: abdominal free fluid.	Norepinephrine, vasopressin, midodrine, heparin, corticosteroids	Discharged after 13 days
Patient 2: 50, male, African American, Florida	None	Poor oral intake, chest pressure, palpitations, diaphoresis x 3 days. Hemodynamically unstable on admission.	No/Testing unknown	PCR (+), Ab (+)	CRP 84 mg/L; D-dimer 2310 ng/mL; ferritin 1919 ng/mL; troponin I 0.48 ng/mL; ALT 440 IU/L; ALC nadir 2500 cells/μL	EKG: atrial fibrillation/flutter with rapid ventricular response, ST segment changes. TTE: ejection fraction 25%–30% with global hypokinesis. CXR: small pleural effusions.	Remdesivir, corticosteroids	Discharged after 17 days
Patient 3: 46, male, African American, Florida	Obesity, chronic right lower extremity pain	Malaise, bilateral tinnitus, chest pain, and vomiting x 4 days. Hypotensive and mildly hypoxemic on admission.	Yes/Testing unknown	PCR (-), Ab (+)	CRP 217 mg/L; D-dimer 3790 ng/mL; ferritin >100,000 ng/mL; troponin I 2.5 ng/mL; IL-6 1412 pg/mL; ALT >10,000 IU/L; ALC nadir 400 cells/μL	EKG: ST-T segment changes. CT chest: dependent ground glass opacities. CT abdomen: hepatic steatosis.	Vasopressors, tocilizumab x 1, heparin	Deceased
Patient 4: 21, male, African American, Louisiana	Obesity	Fever, cough, nausea, vomiting, lymphadenopathy x 6 days.	No/Testing unknown	PCR (-), Ab (+)	CRP 318 mg/L; D-dimer 1760 ng/mL; ferritin 4400 ng/mL; troponin T 0.65 ng/mL; IL-6 7 pg/mL; ATL 279 IU/L; ALC nadir 700 cells/μL	TTE: severely decreased ejection fraction, mild mitral regurgitation, right ventricular dysfunction, coronary artery dilatation. CT chest: ground glass opacities and atelectasis.	ASA, corticosteroids, IVIG x 1	Discharged after 6 days
Patient 5: 33, male, African American, Georgia	Obesity, HTN, depression	Fever, chest pain, abdominal pain, diarrhea, dark urine x 4 days.	Yes/PCR (+) 41 days earlier	PCR (+), Ab (+)	CRP 182 mg/L; D-dimer 275 ng/mL; ferritin 375 ng/mL; troponin I 1.8 ng/mL; IL-6 74.3 pg/mL; ALT 30 IU/L; ALC nadir 2070 cells/μL	CT chest: atelectasis. CT abdomen/pelvis: normal. TTE: mitral and tricuspid regurgitation.	Anticoagulation	Discharged after 5 days
Patient 6: 22, female, African American, New York	None	Fever, chills, throat pain, odynophagia x 2 days.	No/Testing unknown	PCR (+), Ab (+)	CRP 355 mg/L; D-dimer 1882 ng/mL; ferritin 378 ng/mL; troponin T 0.06 ng/mL; IL-6 34.8 pg/mL; ALT 119 U/L; ALC nadir 360 cells/μL	CT neck: retropharyngeal and parapharyngeal edema. EKG: intermittent complete heart block with narrow junctional escape without hemodynamic compromise. TTE: ejection fraction 50%. CXR: dense bilateral lower lobe air-space disease.	Phenylephrine, anticoagulation, corticosteroids	Discharged after 19 days
Patient 7: 21, female, African American, New York	Obesity	Fever, fatigue, throat and neck pain, nausea, vomiting x 1 day.	Yes/PCR (+) 25 days earlier	PCR (+), Ab (+)	CRP 319 mg/L; D-dimer 713 ng/mL; ferritin 351 ng/mL; troponin T 0.04 ng/mL; IL-6 56.2 pg/mL; ALT 160 IU/L; ALC nadir 260 cells/μL	CT neck: bilateral supraclavicular and cervical lymphadenopathy with no discrete abscess or collection. CT chest: bilateral patchy ground-glass opacities, pleural effusion. TTE: mild to moderate diffuse left ventricular hypokinesis. Mild to moderate decreased left ventricular ejection fraction (40%). Small posterior pericardial effusion. Mild tricuspid and mitral valve regurgitation.	Dobutamine, heparin, ASA x1, corticosteroids x2	Discharged after 12 days
Patient 8: 47, female, African American, New York	None	Weakness, sore throat, shortness of breath, decreased exercise tolerance x 3 days.	Yes/Testing unknown	PCR (+), Ab testing not performed	CRP 485 mg/L; D-dimer 1365 ng/mL; ferritin 948 ng/mL; troponin T 0.24 ng/mL; ALT 45 U/L; ALC nadir 1980 cells/μL	EKG: first degree AV block and nonspecific T-wave abnormalities. TTE: borderline left ventricular ejection fraction (55%).	Heparin, convalescent plasma	Discharged after 8 days
Patient 9: 42, male, Asian, New York	Obesity	Fever, shortness of breath, cough, diarrhea, poor appetite, dysuria x 5 days.	Yes/PCR (+) 37 days earlier	PCR (-), Ab testing not performed	CRP 387 mg/L; D-dimer 3519 ng/mL; ferritin 7529 ng/mL; troponin T 0.60 ng/mL; ALT 66 U/L; ALC nadir 1740 cells/μL	TEE: mildly dilated left ventricle, moderately dilated right ventricle, moderate biventricular hypokinesis, moderately decreased left ventricular ejection fraction (35%). CXR: bilateral lower lobe opacities/airspace disease.	Vasopressors, anticoagulation, corticosteroids	Discharged after 9 days

**Abbreviations:** Ab = antibody; ALC = absolute lymphocyte count; ALT = alanine aminotransferase; ASA = aspirin; CRP = C-reactive protein; CT = computed tomography; CXR = chest radiograph; EKG = electrocardiogram; HTN = hypertension; IL-6 = interleukin-6; IVIG = intravenous immunoglobulin; PCR = polymerase chain reaction; TEE = transesophageal echocardiogram; TTE = transthoracic echocardiogram.

* Normal ranges for laboratory studies: ALC 1000–4000 cells/μL; ALT 5–30 IU/L; CRP 0–10 mg/L; D-dimer <500 ng/mL; ferritin 12–300 ng/mL (men), 12–150 ng/mL (women); IL-6 ≤1.8 pg/mL; troponin I <0.03 ng/mL; troponin T < 0.1 ng/mL.

**TABLE 2:** Demographics, clinical features, treatments, and outcomes of seven adults reported in published literature with multisystem inflammatory syndrome (MIS) associated with SARS-CoV-2 infection — United Kingdom and United States, March–August 2020

Age (yrs), sex, race/ethnicity, location	Underlying medical conditions	Clinical signs/symptoms	Previous respiratory illness/SARS-CoV-2 testing	SARS-CoV-2 testing at time of MIS-A admission	Laboratory studies (peak)*	Imaging/Other diagnostic studies	Treatments	Outcome and length of stay
Patient 10^†^: 36, female, Hispanic, New York	None	Fever, abdominal pain, vomiting, and diarrhea x 7 days; arthralgias and diffuse rash x 2 days. On admission, nonexudative conjunctivitis, mucositis, edema of bilateral hands and feet, palmar erythema, diffuse maculopapular rash, and cervical lymphadenopathy.	No/Not tested	PCR (+), Ab (+)	CRP 300 mg/L; D-dimer 652 ng/mL; ferritin 684 ng/mL; troponin I 0.07 ng/mL; ALT 116 IU/L; ALC nadir 900 cells/μL	TTE: moderate tricuspid regurgitation, pericardial effusion. CT chest: right pleural effusion. Ultrasound: gallbladder wall edema.	ASA, IVIG x1, corticosteroids	Discharged after 7 days
Patient 11^§^: 45, male, Hispanic, New York	None	Fever, sore throat, diarrhea, lower extremity pain, and diffuse rash x 6 days. On admission, hypotensive and tachycardic with nonexudative conjunctivitis, periorbital edema, mucositis, unilateral cervical lymphadenopathy, and diffuse exanthem.	No/Not tested	PCR (+), Ab testing not performed	CRP 547 mg/L; D-dimer 2977 ng/mL; ferritin 21,196 ng/mL; troponin 8.1 ng/mL; IL-6 117 pg/mL; ALT 133 IU/L; ALC nadir 700 cells/μL	EKG: ST elevations in anterolateral leads. TTE: ejection fraction 40% with global hypokinesis. CT head/neck: pre-septal edema. Slit lamp: uveitis.	Heparin, corticosteroids, IVIG x 2, Tocilizumab x 1	Discharged after 9 days
Patient 12^¶^: 44, female, Hispanic, Massachusetts	GERD, mild obstructive sleep apnea, depression	Chills, sore throat, cough, myalgias x 2 days (8 days before admission); followed by diarrhea and back pain x 3 days; followed by pleuritic chest pain and dyspnea. Admitted with profound cardiogenic shock.	Yes/Not tested	PCR (+), Ab testing not performed	CRP 141 mg/L; D-dimer 8691 ng/mL; ferritin 2564 ng/mL; hs-Trop T 1810 ng/L; IL-6 53.3 pg/mL; ALT 242 IU/L; ALC nadir 670 cells/μL	EKG: submillimeter ST-segment elevation in leads I/aVL, low QRS voltage. TTE: severely depressed left ventricular function, trace pericardial effusion. CT chest: mild ground glass opacities bilateral lung fields. CT abdomen/pelvis: small amount of ascites, periportal edema.	Norepinephrine, dobutamine, vasopressin, milrinone, IVIG x 5 days, ECMO to LVAD and RVAD.	Discharged to rehabilitation facility after 18 days; home 7 days later
Patient 13**: 21, male, African origin, United Kingdom	None	Fever, headache, and abdominal pain x 6 days; transient palmar rash. Hypotensive on admission with nonexudative conjunctivitis, mucositis, cervical lymphadenopathy.	No/Not tested	PCR (-), Ab (+)	CRP 338 mg/L; D-dimer 4260 ng/mL; ferritin 1249 ng/mL; troponin T 3.3 ng/mL; ALT 330 IU/L; ALC nadir 390 cells/μL	CT abdomen/pelvis: mesenteric adenopathy and ileitis. EKG: sinus tachycardia. CT chest: normal. TTE: normal. CT coronary angiogram: normal.	ASA, corticosteroids, IVIG x 1	Discharged after 8 days
Patient 14^††^: 31, female, African American, Louisiana	Obesity, HTN, diabetes mellitus type 2	Fever x 1 day, throbbing neck pain, nausea, vomiting.	Yes/PCR (+) 14 days before admission	PCR (-), Ab testing not performed	CRP 580 mg/L; D-dimer 453 ng/mL; ferritin 793 ng/mL; ALT 52 IU/L; ALC nadir 2120 cells/μL	Pathology: small-vessel cardiac vasculitis; new pulmonary thrombi in a background of otherwise reparative changes in the lungs. CT head/neck: bilateral enlarged parotid glands. CT chest: interval improvement of bibasilar ground-glass opacities with cervical and anterior mediastinal lymphadenopathy.	CPR	Deceased at admission (ventricular fibrillation)
Patient 15^§§^: 25, female, Hispanic, Georgia	None	Fever, weakness, and shortness of breath x 7 days; followed by sore throat, mild cough, vomiting, and diarrhea. Hypotensive on admission with conjunctivitis, mucositis, cervical lymphadenopathy.	No/Not tested	PCR (+), Ab (+)	CRP 90 mg/L; D-dimer 1918 ng/mL; ferritin 798 ng/mL; troponin I 0.06 ng/mL; ALT 25 IU/L, ALC nadir 1150 cells/μL	TTE: moderate to severely reduced right-sided ventricular dysfunction, flattened interventricular septum in systole consistent with right ventricular pressure overload. EKG: right axis deviation. CT chest: scattered patchy ground glass opacities and peripheral consolidation, small bilateral pleural effusions with adjacent atelectasis; mild enlargement of the main pulmonary artery without pulmonary embolus. CT abdomen/ pelvis: mild peripancreatic fat stranding, nonspecific bilateral perinephric fat stranding.	ASA, IVIG x 2, vasopressors	Discharged after 5 days
Patient 16^¶¶^: 38, female, Hispanic, Texas	None	Fever, occipital headache, conjunctival injection, odynophagia, mucositis, glossitis shortness of breath, vomiting, polyarthralgia, and rash x 5 days.	Yes/PCR (+) 28 days earlier	PCR (+), Ab (+)	CRP 217 mg/L; D-dimer 1250 ng/mL; ferritin 196 ng/mL; troponin I <0.03 ng/mL; ALT 126 IU/L; ALC nadir 120 cells/μL	TTE: trace pericardial effusion, elevated pulmonary artery pressure (46–51 mmHg), normal left ventricular ejection fraction, no coronary artery abnormalities. CT chest/abdomen/pelvis: no pulmonary emboli, right upper lobe perihilar ground-glass opacities, septal and bronchial wall thickening, bilateral small-to-moderate pleural effusions.	ASA, corticosteroids, IVIG x 2	Discharged after 7 days

**Abbreviations:** Ab = antibody; ALC = absolute lymphocyte count; ALT = alanine aminotransferase; ASA = aspirin; CPR = cardiopulmonary resuscitation; CRP = C-reactive protein; CT = computed tomography; ECMO = extracorporeal membrane oxygenation; EKG = electrocardiogram; GERD = gastroesophageal reflux disease; hs-Trop T = high sensitivity troponin T; HTN = hypertension; IL-6 = interleukin-6; IVIG = intravenous immunoglobulin; LVAD = left ventricular assist device; PCR = polymerase chain reaction; RVAD = right ventricular assist device; TTE = transthoracic echocardiogram.

* Normal ranges for laboratory studies: ALC 1000–4000 cells/μL; ALT 5–30 IU/L; CRP 0–10 mg/L; D-dimer <500 ng/mL; Ferritin 12–300 ng/mL (men), 12–150 ng/mL (women); hs-Trop T 0–9 ng/L IL-6 ≤1.8 pg/mL; troponin I <0.03 ng/mL; troponin T < 0.1 ng/mL.

^†^ https://www.sciencedirect.com/science/article/pii/S0735675720305428?via%3Dihub.

**^§^** https://www.thelancet.com/pdfs/journals/lancet/PIIS0140-6736(20)31526-9.pdf.

**^¶^** https://www.nejm.org/doi/10.1056/NEJMcpc2004975.

** https://www.sciencedirect.com/science/article/pii/S2665991320302344?via%3Dihub.

^††^ https://www.acpjournals.org/doi/10.7326/L20-0882.

^§§^ https://bmcinfectdis.biomedcentral.com/articles/10.1186/s12879-020-05439-z.

**^¶¶^** https://ard.bmj.com/content/early/2020/09/25/annrheumdis-2020-218959.

**Initial signs and symptoms.** Twelve of 16 patients had fever (≥100.4°F [38.0°C] for ≥24 hours or report of subjective fever lasting ≥24 hours) at the time of presentation. Six patients were initially evaluated for possible cardiac symptoms such as chest pain or palpitations; all 16 had evidence of cardiac effects, including electrocardiogram abnormalities such as arrhythmias, elevated troponin levels, or echocardiographic evidence of left or right ventricular dysfunction. Thirteen patients had gastrointestinal symptoms on admission; five had dermatologic manifestations on admission, including three with mucositis. Despite minimal respiratory symptoms, 10 patients had pulmonary ground glass opacities, and six had pleural effusions identified on chest imaging. 

**Inflammatory markers.** All patients had markedly elevated laboratory markers of inflammation, including CRP (range of peak values = 84–580 mg/L; upper limit of normal [ULN] = 10 mg/L) and ferritin (196 to >100,000 ng/mL; ULN = 150 ng/mL for women, 300 ng/mL for men), as well as markers of coagulopathy including D-dimer (275–8691 ng/mL; ULN = 500 ng/mL). Ten patients had absolute lymphocyte counts lower than normal range (range of nadir values 120–2120 cells/μL; lower limit of normal = 1000 cells/μL).

**SARS-CoV-2 test results.** Ten patients received positive SARS-CoV-2 PCR test results at the time of initial assessment for MIS-A, seven of whom also had serologic evidence of infection (positive antibody test results) at that time. Six patients received negative SARS-CoV-2 PCR test results; of those, four had positive anti-SARS-CoV-2 antibody test results when first evaluated. Two patients had positive SARS-CoV-2 PCR test results 14 and 37 days before admission, negative PCR results at the time of admission, and no known antibody testing. Three additional patients had positive SARS-CoV-2 PCR test results 25–41 days before admission and continued positive PCR test results at the time of admission.

**Treatment.** Seven patients were treated with intravenous immunoglobulin, 10 with corticosteroids, and two with the interleukin-6 inhibitor, tocilizumab. Ten patients required intensive care; seven required inotropes or vasopressors, and one required mechanical circulatory support (extracorporeal membrane oxygenation followed by temporary left and right ventricular assist devices). Three patients required endotracheal intubation and mechanical ventilation, and two patients died.

## Published Case Series

Three published case series were identified describing adult patients with manifestations consistent with MIS-A (*4*–*6*). One series describes seven previously healthy, young adult men aged 20–42 years who experienced mixed cardiogenic and vasoplegic shock and hyperinflammation along with high SARS-CoV-2 immunoglobulin G antibody titers indicating active or previous infection (*4*). Two of the patients identified as African American, two as Hispanic, two as Middle Eastern, and one as White. Four of the seven patients had negative PCR test results for SARS-CoV-2 at the time of admission, all had markedly elevated inflammatory markers and required inotropes or vasopressors, and three required intraaortic balloon pumps. All were treated with corticosteroids and therapeutic anticoagulation. All seven patients recovered and were discharged home after 7 to 18 days of hospitalization with improved cardiovascular function.

A second case series describes two patients aged 21 and 50 years who came to medical attention because of large-vessel strokes associated with positive SARS-CoV-2 tests (*5*). Information on race/ethnicity of these patients was not reported. These patients had elevated inflammatory markers and minimal respiratory symptoms, consistent with MIS-A. The authors proposed endothelial dysfunction and coagulopathy related to SARS-CoV-2 infection as potential etiologies. Incidence of large-vessel stroke among young adults during this same time the previous year was statistically significantly lower (*5*).

A third case series describes the pathologic findings of endothelialitis and complement deposition in the vessels of two patients with illness resembling MIS-A (cardiac dysfunction, abdominal signs and symptoms, and rash) associated with positive SARS-CoV-2 test results (*6*). Information on race/ethnicity of these patients was not reported. One of these two patients had no underlying medical conditions and recovered; the other had multiple underlying conditions at higher risk for severe COVID-19 and died hours after seeking care. Pathologic findings in this case series were similar to autopsy findings for those of patient 14 (Table 2).

## Discussion

Findings indicate that adult patients of all ages with current or previous SARS-CoV-2 infection can develop a hyperinflammatory syndrome resembling MIS-C. Although hyperinflammation and extrapulmonary organ dysfunction have been described in hospitalized adults with severe COVID-19, these conditions are generally accompanied by respiratory failure (*7*). In contrast, the patients described here had minimal respiratory symptoms, hypoxemia, or radiographic abnormalities in accordance with the working case definition, which was meant to distinguish MIS-A from severe COVID-19; only eight of 16 patients had any documented respiratory symptoms before onset of MIS-A.

The pathophysiology of MIS in both children and adults is currently unknown. Eight of 27 (30%) adults described in this report and 45% of 440 children with MIS-C reported to CDC through July 29, 2020, (*1*) had negative PCR and positive SARS-CoV-2 antibody test results, suggesting MIS-A and MIS-C might represent postinfectious processes. However, in some patients, persistent infection outside the upper respiratory tract is possible; SARS-CoV-2 has been identified in multiple organs including the heart, liver, brain, kidneys, and gastrointestinal tract (*7*). Additional proposed mechanisms for extrapulmonary dysfunction in COVID-19 include endothelial damage and thromboinflammation, dysregulated immune responses, and dysregulation of the renin-angiotensin-aldosterone system (*7*).

The interval between infection and development of MIS-A is unclear, adding to uncertainty regarding whether MIS-A represents a manifestation of acute infection or an entirely postacute phenomenon. In patients with COVID-19, dyspnea is typically experienced a median of 5–8 days and critical illness 10–12 days after onset of symptoms.^§^ In patients who reported typical COVID-19 symptoms before MIS-A onset, MIS-A was experienced approximately 2–5 weeks later. However, eight MIS-A patients reported no preceding respiratory symptoms, making it difficult to estimate when initial infection occurred.

Given the high proportion of MIS-C patients with negative PCR testing, clinical guidelines recommend the use of both antibody and viral testing to assist with diagnosis (*8*–*10*). In patients with atypical or late manifestations of SARS-CoV-2 infection, including MIS-A, positive antibody results might be crucial to augment clinical recognition of this condition and guide treatment. In addition, the use of a panel of laboratory tests for inflammation, hypercoagulability, and organ damage (e.g., CRP, ferritin, D-dimer, cardiac enzymes, liver enzymes, and creatinine) might assist in the early identification and management of this COVID-19–associated condition.

All but one of the patients with MIS-A described in this report belonged to racial or ethnic minority groups. Long-standing health and social inequities have resulted in increased risk for infection and severe outcomes from COVID-19 in communities of color.^¶^ MIS-C has also been reported disproportionately in these communities (*1*). Because patients described in this review represent a convenience sample from a small number of jurisdictions, conclusions cannot be made regarding the true burden or determinants of MIS-A in different groups; further research is needed.

The majority (24 of 27) of patients with MIS-A survived, similar to those with MIS-C, associated with receiving care in acute, often intensive, health care settings. Because of the potential therapies that might benefit these patients as described in these case reports, clinicians should consider MIS-A within a broader differential diagnosis when caring for adult patients with clinical and laboratory findings consistent with the working MIS-A case definition.

The findings in this report are subject to at least three limitations. First, cases described here were voluntarily reported or published and therefore are not representative of the true clinical spectrum or racial/ethnic distribution of this emerging syndrome. Additional cases might not have been reported or published; others might have remained unrecognized because of absence of COVID-like symptoms, lack of antibody testing, or negative test results. Second, the working case definition excludes patients with severe respiratory dysfunction to distinguish MIS-A from severe COVID-19; however, the two conditions might overlap in some cases. Finally, the working case definition for this syndrome is potentially nonspecific, and some patients with other disease processes might have been misclassified as having MIS-A.

Clinicians and health departments should consider MIS-A in adults with signs and symptoms compatible with the current working MIS-A case definition. Antibody testing for SARS-CoV-2 might be needed to confirm previous COVID-19 infection in patients who do not have positive SARS-CoV-2 PCR or antigen test results. Findings in this convenience sample emphasize the importance of collecting race/ethnicity data on case reports at the jurisdictional level. As with children, it is important that multidisciplinary care be considered to ensure optimal treatment. In the process of learning more from MIS-A cases, the working case definition might need to be revised in order to systematically conduct a call for cases. Further research is needed to understand the pathogenesis and long-term effects of this newly described condition. Ultimately, the recognition of MIS-A reinforces the need for prevention efforts to limit spread of SARS-CoV-2.

**Summary**

**What is already known about this topic?**

Multisystem inflammatory syndrome in children (MIS-C) is a rare but severe complication of SARS-CoV-2 infection in children and adolescents. Since June 2020, several case reports and series have been published reporting a similar multisystem inflammatory syndrome in adults (MIS-A).

**What is added by this report?**

Cases reported to CDC and published case reports and series identify MIS-A in adults, who usually require intensive care and can have fatal outcomes. Antibody testing was required to identify SARS-CoV-2 infection in approximately one third of 27 cases.

**What are the implications for public health practice?**

Clinical suspicion and indicated SARS-CoV-2 testing, including antibody testing, might be needed to recognize and treat adults with MIS-A. Further research is needed to understand the pathogenesis and long-term effects of this condition. Ultimately, the recognition of MIS-A reinforces the need for prevention efforts to limit spread of SARS-CoV-2.

## Footnotes

* Multisystem Inflammatory Syndrome Associated with COVID-19 Case Report Form. https://www.cdc.gov/mis-c/pdfs/hcp/mis-c-form-fillable.pdf.

† Medline (OVID), Embase (OVID), CINAHL (EBSCOHost) and Cochrane Library were searched as primary sources, which were supplemented with searches in the following databases: Global Health, CAB abstracts, PsycInfo, Scopus, PubMed Central, Global Index Medicus, and several preprint databases. Each database was searched using the following terms: novel coronavirus/COVID-19 (multiple iterations) and severe inflammation/multisystem, cardiogenic shock/Kawasaki disease, and adult.

§ https://www.cdc.gov/coronavirus/2019-ncov/hcp/clinical-guidance-management-patients.html.
